# Chromatography and Spectrometry in Food Safety and Pharmaceutical Analysis

**DOI:** 10.3390/molecules31152629

**Published:** 2026-07-28

**Authors:** Małgorzata Dołowy, Josef Jampilek, Katarzyna Bober-Majnusz

**Affiliations:** 1 Department of Analytical Chemistry, Faculty of Pharmaceutical Sciences in Sosnowiec, Medical University of Silesia in Katowice, Jagiellońska 4, 41-200 Sosnowiec, Poland; bober@sum.edu.pl; 2 Department of Analytical Chemistry, Faculty of Natural Sciences, Comenius University, Ilkovicova 6, 842 15 Bratislava, Slovakia; josef.jampilek@gmail.com

## 1. Introduction

**Figure 1:**
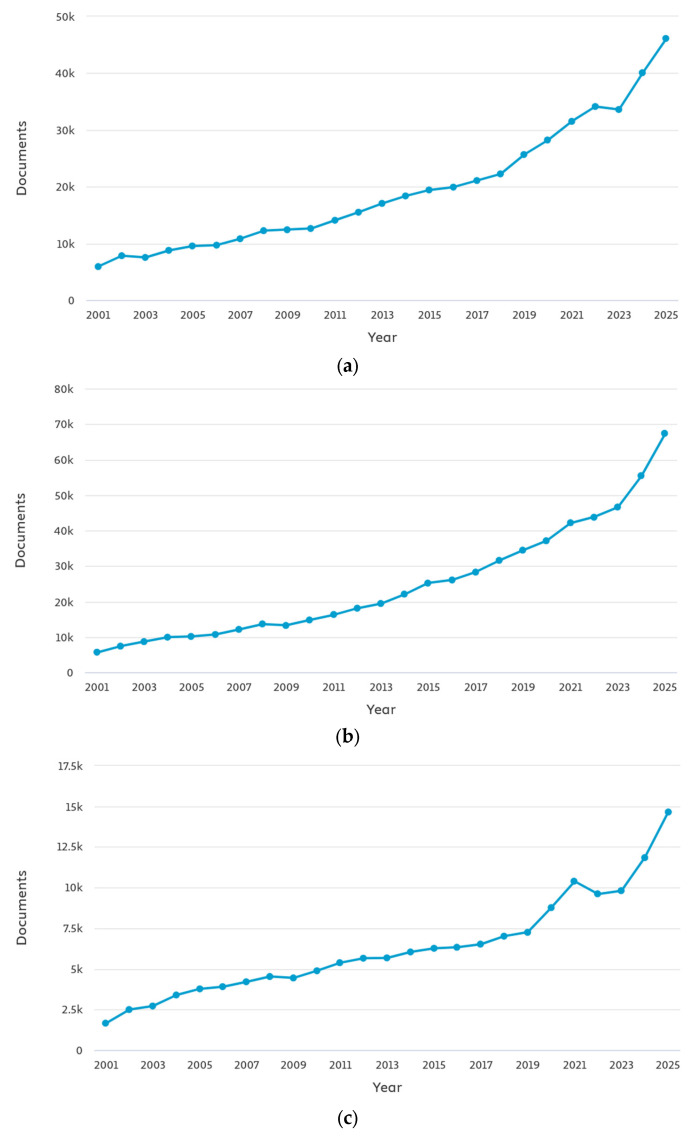
Graphs documenting the number of published documents in the years 2001–2025 in the Scopus database as of 12 June 2026: (**a**) keywords “chromatography AND spectrometry”; (**b**) keyword “food analysis”; (**c**) keyword “pharmaceutical analysis”.

**Figure 2:**
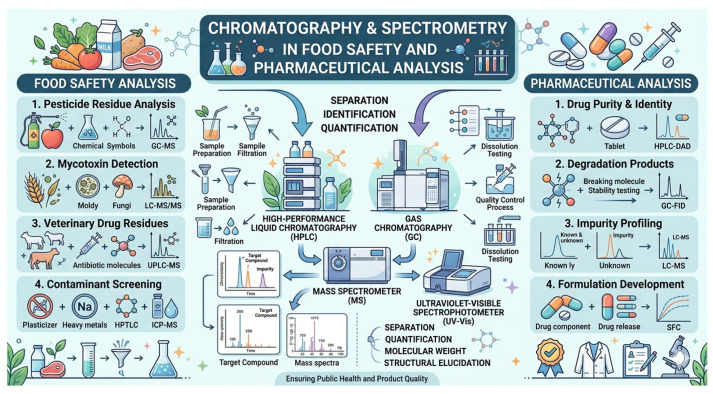
Scheme illustrating versatile use of chromatography and spectrometry in food and pharmaceutical analyses.

Liquid chromatography—including thin-layer chromatography (TLC) and high-performance liquid chromatography (HPLC/LC)—and gas chromatography (GC) are fundamental separation techniques in analytical chemistry, used for the separation, identification, and quantification of individual components in a mixture across a wide range of applications, from biochemistry, pharmaceutical, food, and environmental research to forensic analysis [1,2,3,4,5]. The combination of chromatography and mass spectrometry (MS) represents the “gold standard” in modern analytical chemistry. Together, these techniques allow for the identification and quantification of substances at extremely low concentrations, even in very complex mixtures such as biological or food samples [6,7,8,9,10,11,12]. The graphs in Figure 1 show the progression of published works on the applications of chromatographic methods in food and drug analysis during the first quarter of the new millennium. Based on these data, it can be said that chromatography and spectrometry are completely universal analytical techniques and extremely useful tools that are applicable in food, pharmaceutical, and environmental analysis, and capable of analyzing both simple and complex samples/matrices, as shown in Figure 2.

In the food industry, chromatographic systems/techniques are applied to consumer protection and quality assurance. Highly sensitive methods such as liquid chromatography–tandem mass spectrometry (LC-MS/MS) allow for contaminant analysis and residue monitoring, i.e., the detection of hundreds of different pesticides and other agrochemicals in a single sample of fruit, vegetables, and other foods, even trace amounts below the limits set by legislation, or the detection of mycotoxins, heavy metals, or other substances migrating from packaging materials (e.g., phthalates) [7,13,14,15,16,17,18,19,20,21]. It is important to mention that chromatography also helps to verify the authenticity of food and detect its adulteration, for example, the dilution of olive oil, the addition of synthetic sweeteners to honey, or the substitution of a declared type of meat or fish [22,23,24,25].

Pharmaceutical analysis is a field of study concerned with the qualitative and quantitative (identification, purity determination) evaluation of active pharmaceutical ingredients (APIs), excipients, and final dosage forms [26,27,28]. This field can be understood as part of medicinal (pharmaceutical) chemistry or as a specific application of analytical chemistry. In any case, separation methods—specifically, chromatography with MS detection—have become an essential part of the process for ensuring the safety and efficacy of drugs in pharmaceutical development and manufacturing [29,30,31,32,33,34]. In addition, LC-MS/MS is commonly used in bioanalysis to monitor drug (and their metabolites) levels in the blood or tissues of patients during clinical trials and for proper treatment adjustment. Chromatographic methods are also used to monitor how a drug substance degrades over time due to temperature, humidity, or light, which directly determines the expiration date on the drug packaging [8,35,36,37]. In this context, it is worth mentioning the presence of LC-MS/MS methods among “omics” techniques [10,38,39,40,41,42,43,44] and the use of chromatographic methods in the fight against counterfeit medicines, as they allow us not only to determine whether a medicine contains the declared active substance, but also in what quantity and whether or not it contains dangerous additives or impurities [45,46].

The purpose of this Special Issue is to present new insights regarding the application of chromatographic and spectrometric methods for the accurate separation, identification, and quantification of various natural and synthetic bioactive compounds as potential drugs and food ingredients. This Issue received five original scientific papers.

## 2. An Overview of Published Articles

This Special Issue for the journal Molecules; https://www.mdpi.com/journal/molecules/special_issues/0D5YVNY9ED (accessed on 23 August 2023) belongs to the section “Analytical Chemistry”. Within this Issue, five original articles were published. These papers were authored by scientists from Saudi Arabia, South Africa, Poland, China, Malaysia, Belgium, Malta, and India.

The aim of the study conducted by Attimarad et al. (Contribution 1) was to develop a rapid, simple, and effective HPLC method for the simultaneous determination of metoprolol, telmisartan, and amlodipine in a product (a novel combination of three active substances) intended for the treatment of hypertension. The study employed a Box–Behnken design (BBD) to predict and optimize chromatographic conditions for the proposed analytical method. The BBD model enabled the rapid and cost-effective selection of appropriate analysis parameters, such as a C18 HPLC column, a mobile phase consisting of a mixture of acetonitrile (45%) and phosphate buffer (20 mM, pH 5.8; 55%) at a flow rate of 1.1 mL/min, and detection at a wavelength of 230 nm. The method enables the quantitative determination of metoprolol, telmisartan, and amlodipine with limits of quantitation (LOQ) at 3.68, 7.88, and 0.33 µg/mL, respectively. The experimental model was verified through testing under optimized chromatographic conditions. The results obtained—including retention time (with a relative error of less than 5%)—confirmed the suitability of the established design space for predicting optimal experimental conditions. Method validation, conducted in accordance with ICH (International Council for Harmonisation) guidelines regarding linearity, limits of detection and quantitation, accuracy, precision, and robustness, confirmed the suitability of the developed HPLC procedure for its intended purpose. Furthermore, the application of both the AGREE (Analytical Greenness) metric and the principles of “white analytical chemistry” demonstrated the eco-friendly nature of the described HPLC method. These attributes confirmed the suitability of the newly developed method for the routine analysis of preparations containing the three active substances under investigation.

Nisterenko et al. (Contribution 2) published results confirming the utility of combined methods—specifically, theoretical (in silico) and experimental approaches such as liquid chromatography—during the early stages of drug discovery. The authors employed a chromatographic technique to determine experimental lipophilicity parameters for a series of 26 newly synthesized ipsapirone derivatives, which are being investigated as potential therapeutic agents for symptoms of depression or schizophrenia. RP-HPLC analyses were performed using a C18 column and a diode-array detector (DAD). The mobile phases consisted of a mixture of acetonitrile (eluent B) and one of the following aqueous solutions (eluent A): acetic acid (pH 2.6), ammonium acetate (pH 7.4), or ammonium acetate (pH 10.5). In parallel, to assess the passive diffusion of the molecule across the blood–brain barrier (BBB), parameters describing its lipophilicity—expressed as the logarithm of the partition coefficient (as MLogP, ALogP, LogP99, LogPcons (AlvaDesc), LogPChemicalize, iLogP, XLogP3, WLogP, LogPcons (SwissADME), Silicos-IT LogP)—were calculated using AlvaDesc (version 2.0.10, Alvascience, Lecco, Italy) and SwissADME software (web application: http://www.swissadme.ch, accessed on 1 February 2024), based on the SMILES formula. The HPLC method employing immobilized artificial membrane (IAM) columns also enabled the determination of the affinity of the tested compounds for phospholipids (CHI_IAM_ descriptor) and human plasma proteins (expressed as logK_HSA_). The use of columns modified with plasma proteins, such as human serum albumin (HSA), allowed for the estimation of the extent of plasma protein binding (PPB), a parameter influencing drug pharmacokinetics, including distribution, half-life, and clearance. Cluster analysis (CA) was performed using Ward’s agglomerative method and the Euclidean distance measure, employing a custom R script. For the regression analysis within the QSRR (quantitative structure–retention relationship) study, a genetic algorithm (GA) and multiple linear regression (MLR) were applied, incorporating both theoretical descriptors and experimentally determined properties. It was demonstrated that QSRR modeling, based on experimental and computational data, enabled the identification of key molecular descriptors influencing lipophilicity, phospholipophilicity, and HSA binding of the studied compounds. It was stated that integrating experimental data with predictive models of plasma protein binding improved model performance, and highlighted the importance of chromatographic assessments.

The third article published in this Special Issue (Contribution 3) focused on the development of a sensitive and rapid HPLC method coupled with tandem mass spectrometry (HPLC-MS/MS) for the determination of an innovative drug—vepdegestrant—in biological samples, specifically mouse and rat plasma. The drug under investigation, known as ARV-471, is a novel proteolysis-targeting chimera (a PROTAC-type drug) that acts as an estrogen receptor alpha (ERα) degrader in breast cancer patients. For the quantitative determination of vepdegestrant, a C18 column and a mobile phase consisting of an aqueous ammonium formate solution and acetonitrile (in a 30:70 *v*/*v*) was used at a flow rate of 0.3 mL/min. Chromatographic analyses employed a QTrap system coupled with an electrospray ionization (ESI) interface, operating in positive ionization mode. Bavdegalutamide (designated as ARV-110) was used as the internal standard. The method was successfully validated in accordance with current guidelines for selectivity, accuracy, precision, stability, robustness, and sensitivity. Validation results for all parameters—including the matrix effect—confirmed compliance with the acceptance criteria established by the European Medicines Agency (EMA) and the US Food and Drug Administration (US FDA). The developed method demonstrated linearity in the range of 1 to 1000 ng/mL in mouse and rat plasma. The lower limit of quantitation (LLQ) was 3 ng/mL. The physicochemical stability of vepdegestrant was determined using buffers of varying pH levels (2.0–10.0), phosphate-buffered saline (pH 7.4), and plasma and liver microsomes from mice, rats, dogs, and humans. The developed analytical method can be successfully used to conduct pharmacokinetic (PK) studies of the drug. The following pharmacokinetic parameters were determined: T_max_—time to reach maximum plasma concentration (C_max_); T_1/2_—elimination half-life; CL—systemic clearance; V_ss_—steady-state volume of distribution; MRT—mean residence time; BA—bioavailability. Pharmacokinetic studies conducted in mice and rats demonstrated that vepdegestant exhibited low-to-moderate clearance and a moderate volume of distribution, as well as low-to-moderate bioavailability resulting from limited absorption.

The fourth paper published in the Special Issue (Contribution 4) described the development of a new HPLC-UV method for the determination of isorhapontigenin—a dietary derivative of resveratrol known as trans-3,5,4′-trihydroxy-3′-methoxystilbene—which exhibits various health-promoting properties. Chromatographic analysis was performed using a C18 column and a mobile phase consisting of acetonitrile and formic acid (0.1% *v*/*v*), with a flow rate of 1.5 mL/min at a temperature of 50 °C. Measurements were conducted at a wavelength of 325 nm. The developed HPLC method for the quantitative determination of isorhapontigenin in various mouse biological matrices was validated in accordance with EMA and US FDA guidelines. The validation parameters demonstrated that the proposed method is selective, precise, accurate, and stable under the employed chromatographic conditions, and enables the determination of isorhapontigenin in biological samples (i.e., plasma and tissues) with the lowest limit of quantitation set at 15 ng/mL. The main advantage of this procedure is the minimal matrix effect. For the plasma samples and tissue homogenates analyzed, the preparation procedure is limited to simple, direct protein precipitation. This successfully validated HPLC-UV method was employed in comprehensive studies on the biodistribution of isorhapontigenin in mice. The authors confirmed that isorhapontigenin exhibits bioavailability in target organs and represents a promising subject for further research into nutraceuticals. The authors suggested that the developed HPLC method can serve as a useful tool in the design of compounds belonging to this group, namely nutraceuticals.

The final paper (Contribution 5) concerns the DECAF method—a technique for the quantitative determination of therapeutic oligonucleotides based on the deconvolution of extracted-ion chromatograms. It describes an innovative methodology based on a simple and computationally efficient procedure for deconvolving overlapping isotopic patterns directly from MS1 (mass spectrum 1) data. The method models experimental isotope distributions as mixtures of theoretical templates across retention time, generating deconvoluted ion chromatograms whose peak areas accurately reflect the contributions of individual components. LC-MS analysis was conducted on the UPLC peptide BEH C18 column at 75 °C. A mixture consisting of triethylamine and 1,1,1,3,3,3-hexafluoro-propan-2-ol in water (mobile phase A) and a methanol–acetonitrile mixture (70/30, *v*/*v*) in mobile phase B at a flow rate of 0.3 mL/min were used in this study. MS studies were performed in negative ion mode using an electrospray ionization source. This study confirmed that DECAF is a promising tool for the quantification of biomolecules that co-elute as well as overlap in MS spectra, including therapeutic oligonucleotides.

## 3. Conclusions

This collection of five papers focuses on the applications of chromatography and spectrometry in the fields of food safety and pharmaceutical analysis. All the published studies document the pivotal role of liquid chromatography—combined with modern techniques such as mass spectrometry (both single and tandem)—in the analysis of food constituents and active pharmaceutical ingredients. The results indicate that the application of a Box–Behnken experimental design enabled the development of a rapid HPLC method for the quantitative determination of active substances in new, complex pharmaceutical formulations used to treat hypertension, thereby streamlining quality control for drugs of this class. The works also confirmed that a properly validated LC-MS/MS (HPLC-MS/MS) analytical method—meeting EMA and US FDA guidelines and acceptance criteria—can be successfully employed not only for concentration determination but also for pharmacokinetic profiling in biological samples for other new drugs introduced for cancer therapy, such as vepdegestant (a member of the class of chimeric molecules designed for targeted proteolysis, known as PROTACs). Other studies demonstrate that combining computational methods with HPLC-DAD is crucial for analyzing key molecular descriptors—such as lipophilicity, phospholipid affinity, and HSA binding capacity—of new ipsapirone derivatives considered as potential bioactive molecules. These results highlight the utility of relatively new tools, such as deconvoluted extracted-ion chromatograms, in the determination, identification, and impurity profiling processes—and thus in the pharmaceutical quality control of therapeutic oligonucleotides—particularly regarding the automated resolution of overlapping signals from different compounds in LC–MS datasets. It has been determined that among the various HPLC systems, ultraviolet (UV) detection can be successfully employed as a bioanalytical technique to assess the oral bioavailability and extensive tissue distribution of isorhapontigenin (trans-3,5,4′-trihydroxy-3′-methoxystilbene—a dietary resveratrol derivative), and is a promising nutraceutical candidate. These results provide key information essential for optimizing its use as a nutraceutical.
